# COVID-19 Vaccine Acceptance, Hesitancy, and Uptake in People with Diabetes in Australia

**DOI:** 10.3390/vaccines12060662

**Published:** 2024-06-16

**Authors:** Holly Wang, Lisa Grech, Jennifer Wong, David Hoffman, Barbora de Courten, Brett Sillars, Mark Savage, Alastair Kwok, Mike Nguyen, Nathan Bain, Daphne Day, Eva Segelov, on behalf of the DIABVACCS Investigators

**Affiliations:** 1 Department of Diabetes, Monash Health, Clayton, VIC 3168, Australiajennifer.wong@monashhealth.org (J.W.); 2 School of Psychology, Faculty of Health, Deakin University, Burwood, VIC 3125, Australia; 3 Department of Medicine, School of Clinical Sciences, Monash University, Clayton, VIC 3168, Australia; 4 Dr David Hoffman, Fairfield, NSW 2165, Australia; 5 School of Health and Biomedical Sciences, RMIT University, Melbourne, VIC 3085, Australia; 6 Department of Endocrinology, Sunshine Coast Hospital and Health Service, Birtinya, QLD 4575, Australia; 7 Department of Endocrinology, Bendigo Health, Bendigo, VIC 3550, Australia; msavage@bendigohealth.org.au; 8 Department of Oncology, Monash Health, Clayton, VIC 3168, Australia; 9 Department of Clinical Research, Faculty of Medicine, University of Bern, 3012 Bern, Switzerland

**Keywords:** COVID-19, diabetes, vaccine hesitancy, vaccination

## Abstract

**Background:** This study explored vaccination hesitancy, diabetes-specific COVID-19 vaccination concerns, and whether they predicted vaccination uptake in people with diabetes. **Methods:** Quantitative, cross-sectional, and predictive approaches were used. An online survey was conducted with people with diabetes attending four Australian health services, using convenience sampling (*n* = 842). The survey data collected included clinico-demographic characteristics, COVID-19 vaccine hesitancy, and attitudes around COVID-19 vaccine confidence and complacency. Clinico-demographic characteristics that predicted vaccination status, vaccine hesitancy, and vaccine-related attitudes were identified using regression analyses. **Results:** Most participants received at least one COVID-19 vaccine dose. Younger age and type 1 diabetes were associated with lower vaccination status, and they were partially mediated through higher vaccine hesitancy. Younger age and English as a dominant language were associated with higher negative attitudes towards speed of vaccine development. **Conclusions:** Despite an overall high vaccination rate, general and diabetes-specific COVID-19 vaccine concerns are a barrier to uptake for some people with diabetes, particularly in those who are younger or have type 1 diabetes. A detailed understanding of concerns for particular subgroups can help tailor information to increase vaccine acceptance, particularly in the context of requiring booster doses.

## 1. Introduction

Diabetes is well established as one of the most common comorbidities of COVID-19 infection [1]. People with diabetes have a three-fold higher risk of severe illness from COVID-19, complications, and mortality [2,3,4]. These poorer outcomes are due to immune dysfunction, increased inflammation, impaired viral clearance, and higher airway glucose concentrations predisposing patients to severe respiratory infections [3]. Furthermore, in people with diabetes, the risk of poor outcomes increases with age, obesity, obstructive sleep apnea, poor glycemic control, and pre-existing micro- and macro-vascular complications [1,3,5]. As such, people with diabetes were prioritized for COVID-19 vaccination in Australia and internationally [6,7,8].

Despite poorer outcomes, people with diabetes have reported COVID-19 vaccine hesitancy due to a fear of side-effects, the speed of development, and safety concerns [9,10,11]. Vaccine hesitancy is “a psychological state of indecisiveness that people may experience when making a decision regarding vaccination” [12] (p. 1639). Vaccine uptake and vaccine hesitancy should be considered as distinct entities. People who are vaccinated may still be hesitant, and conversely, people who are accepting may not yet be vaccinated. Vaccine hesitancy is complex and can be influenced by many factors, such as vaccine safety concerns, as well as social and political determinants [13,14].

Vaccine hesitancy has been positively associated with higher glycated hemoglobin (HbA1c) and triglyceride levels in people with type 1 diabetes, and to obesity and lower creatinine levels in people with type 2 diabetes [15]. Consistent with COVID-19 vaccine hesitancy in other populations, greater hesitancy in people with diabetes has been shown in those of younger age, female gender, and lower education level [9,16,17,18]. One study was conducted with individuals with at least one chronic health condition, such as cancer and cardiovascular disease, who are more susceptible to COVID-19 infection, severity, and mortality. It found that many individuals reported experiencing COVID-19 vaccine hesitancy, and this hesitancy was attributed to underlying reasons such as vaccine safety and side-effect concerns [18].

International estimates of vaccine hesitancy in people with diabetes vary widely. A systematic review and meta-analysis of seven studies estimated the proportion of people with diabetes who were vaccine hesitant was 28% [19]. Vaccine hesitancy reported in the included studies ranged from as low as 14.2–18.3% in Italy [9,15] to as high as 56.4% in China [10].

Previous research into vaccine hesitancy in people with diabetes has not explored disease-specific beliefs and concerns, which may contribute to hesitancy. The aim of this multi-site Australian study was to understand vaccination hesitancy and disease-specific COVID-19 vaccination beliefs, and whether it predicts vaccine uptake or intent in people with diabetes.

## 2. Materials and Methods

### 2.1. Study Design

A cross-sectional survey was administered at four Australian health organizations, spanning both metropolitan and regional locations. Survey data collection commenced on 30 August 2021 and concluded on 5 October 2021. During this period, COVID-19 vaccine rollout advice and availability and Australian state government COVID-19 restrictions were implemented (Supplementary Figure S1). The Monash Health Human Research Ethics Committee approved the study protocol (reference number: RES-21-0000-364L-76466). Retrospective study registration was completed with the Australian New Zealand Clinical Trials Registry (registration number: ACTRN12621001467820).

### 2.2. Participants and Sampling

Convenience sampling was used to recruit participants. At each participating health organization, adults scheduled to attend a diabetes and/or endocrinology appointment in the upcoming six months were identified and invited. Short Message Service (SMS) was used to distribute the invitations, which included a hyperlink to the participant information and consent. Potential participants provided electronic informed consent to complete the anonymous survey, which was presented in English. Although the survey was hosted electronically using the Qualtrics^®^ survey platform (Seattle, DC, USA), it was available in hard copy upon patient request.

The 44-item survey was developed by a team of clinicians/researchers and consumer representatives. It comprised sociodemographic, clinical, and COVID-19 vaccine hesitancy and beliefs (Supplementary Table S1).

### 2.3. Measures

**Sociodemographic factors:** Gender, age, education, annual household income, whether the participant identifies as indigenous, and whether English was his/her first language.

**Clinical factors:** Diabetes type, diagnosis duration, current treatment, most recent HbA1c%, perceived diabetes management in the past month, and impact of diabetes on daily activities in the past month.

**COVID-19 vaccination status:** COVID-19 vaccine doses that were received at time that survey collection was completed.

**COVID-19 vaccine hesitancy and beliefs:** The 7-item Oxford COVID-19 Vaccine Hesitancy Scale and the 14-item Oxford COVID-19 Vaccine Confidence and Complacency Scale were used to measure vaccine hesitancy and vaccine confidence and complacency attitudes, respectively [17]. The Oxford COVID-19 Vaccine Confidence and Complacency Scale comprised four subscales: attitudes towards the collective importance of a COVID-19 vaccine, beliefs that that the vaccine will be effective in the event of COVID-19 infection, speed of vaccine development concerns, and vaccine side-effect concerns [17] Higher scores suggested higher levels of negativity towards COVID-19 vaccination. The 6-item Disease Influenced Vaccine Acceptance Scale-Six (DIVAS-6) was used to assess the impact of diabetes and its treatment on the views of COVID-19 vaccination. It comprises a summary scale and two subscales that encompass disease complacency and vaccine vulnerability [20]. These three scales use a 5-point Likert scale with an additional unscored “don’t know” option.

### 2.4. Analytic Strategy

Survey responses were analyzed after removing incomplete, duplicate, or ineligible responses. Missing data were not substituted. Items were summed to calculate summary and subscale scores. “Don’t know” responses were excluded in the calculation of scores [17].

Descriptive statistics were used to summarize the sociodemographic and clinical characteristics and summary scale and subscale scores. Due to low observations, some variable categories were merged or removed: (1) for the highest level of education variable, three categories were combined into one—no formal education, primary school, and secondary school; and (2) non-binary and other gender category variables were removed. Vaccinated status was defined as people who had received at least one vaccine dose, whereas those who had received no dose were considered unvaccinated. We also analyzed participants who received two vaccine doses and, where these results differed from those of participants who had received at least one dose, the results have been reported. When describing the proportion of agreement with each individual DIVAS-6 item by vaccination status, the frequencies of participants’ “somewhat agree” and “strongly agree” responses were combined.

Individual linear and logistic regression analyses controlled for time were conducted. Firstly, demographic and disease-related variables and the outcome variable of interest were analyzed using Pearson’s and Spearman’s Rho. If they were significantly correlated with the outcome variable of interest with a correlation strength of r > 0.10 or r < −0.10, then these demographic and disease-related variables were selected for inclusion as independent variables in the regression analyses. Significant individually regressed variables were then entered into a hierarchical multivariable regression with the outcome variable of interest. A mediation analysis was systematically performed for the demographic and disease-related variables (independent variables) that were significantly correlated at r > 0.10 with vaccinated status (dependent variable), using the scale and subscale scores as mediators and time since study commencement as a covariate. A *p*-value < 0.05 threshold for statistical significance was applied to all analyses. The software program SPSS Statistics Version 27.0 (IBM, Armonk, NY, USA) was used to perform all statistical analyses. The PROCESS macro for the SPSS software was used to conduct the mediation analysis, and it involved applying a bootstrapping approach to identify all significant and indirect effect sizes. Our analyses used 10,000 bootstrap samples to derive confidence intervals [21].

The research results were reported in accordance to the Strengthening the Reporting of Observational Studies in Epidemiology (STROBE) Statement [22].

## 3. Results

### 3.1. Participant Characteristics

Of the 5513 patients invited, 914 survey responses were received. After removing 6 duplicate and 66 ineligible responses, 842 eligible survey responses were analyzed. The median age was 58 (IQR 19) years, and there were 378 (44.9%) females. The sociodemographic and clinical characteristics are presented in Table 1. Type 2 diabetes was the most reported diabetes type (557, 66.2%), followed by type 1 diabetes (252, 29.9%) and other/unknown (33, 3.9%). Most participants (525, 62.4%) indicated that they had been diagnosed with diabetes over ten years ago; 827 (98.1%) reported treatment that included either insulin (279, 33.1%), non-insulin agents (184, 21.9%), or a combination (364, 43.2%). An HbA1c reading of 7.0% to 8.5% (53 to 69 mmol/mol) was reported by 334 participants (39.9%), whereas 302 (35.9%) participants self-reported their diabetes management as “good” in the past month. Of the cohort, 246 participants (29.3%) reported that their diabetes did not have an impact on their daily activities in the past four weeks, whereas 52 (6.2%) reported that it impacted their daily activities all the time. Participant characteristics when vaccinated status was defined as two doses are provided in the Supplementary Table S2.

### 3.2. Vaccine Uptake

Most participants (696, 82.7%) had at least one COVID-19 vaccine dose. Older participants and those with type 2 diabetes were more likely to be vaccinated when compared with younger participants or those with type 1 diabetes, respectively (Table 2). On the multivariable analysis, age and type 2 diabetes both remained significantly associated with higher vaccine uptake (Supplementary Table S3).

When vaccinated status was defined as two doses, older age and a diabetes diagnosis of more than 10 years were associated with a higher likelihood of vaccination, when compared with younger age and a diabetes diagnosis of less than 10 years (Supplementary Table S4). Diabetes type was not associated with vaccinated status. On multivariable analysis, younger age, and a diabetes diagnosis duration of 5.1–10 years both remained significantly associated with lower vaccine uptake (Supplementary Table S5).

### 3.3. Vaccine Hesitancy

Younger age was associated with greater COVID-19 vaccine hesitancy on the Oxford COVID-19 Vaccine Hesitancy Scale (Table 3). There was a partially mediated relationship between younger participants or having type 1 diabetes and reduced vaccination through higher vaccine hesitancy when analyzed with any vaccination (one or two), while a fully mediated relationship was seen for the group who had received two vaccinations (Table 4).

### 3.4. Vaccine-Related Attitudes and Beliefs

#### 3.4.1. Summary Scale

Female gender and younger age were related and showed greater negative attitudes related to confidence and complacency of COVID-19 vaccination (Supplementary Table S6). Greater negative vaccine attitudes were reported by participants who perceived diabetes impacted on their daily activities all the time or most of the time, compared to those who reported less frequent impact. Participants whose most recent HbA1c result was unknown reported lower negative vaccine attitudes than those who reported their most recent HbA1c as <7% (53 mmol/mol). In the multivariable analysis, gender, age, and impact of diabetes on daily activities remained significant (Supplementary Table S7). There was a partially mediated relationship with higher age and higher vaccination through more positive attitudes around confidence and complacency of the COVID-19 vaccination for people reporting any number of vaccinations (Table 4). This relationship was fully mediated for people reporting two vaccination doses.

#### 3.4.2. Collective Importance Subscale

Participants who reported English was their non-dominant language reported more positive attitudes towards collective importance of the vaccine, compared with participants who reported English as their dominant language (Supplementary Table S6). In the multivariable analysis, English as a non-dominant language and diabetes type remained significant predictors of collective importance (Supplementary Table S8). There was a partially mediated relationship between having type 1 diabetes with lower vaccine uptake in participants who had received at least one vaccination through lower collective importance (Table 4). There was a fully mediated relationship between being under treatment for diabetes and greater vaccine uptake through higher collective importance.

#### 3.4.3. Beliefs about COVID-19 and Vaccine Subscale

Female gender was associated with a higher subscale score for beliefs about COVID-19 and vaccine, indicating greater negative beliefs about the COVID-19 vaccine’s effectiveness (Supplementary Table S6). A partially mediated relationship between younger age and lower vaccine uptake was found through more negative beliefs about the COVID-19 vaccine’s effectiveness (Table 4). This was significant for groups receiving any vaccinations (one or two) or two vaccinations. There was a partially mediated relationship between having type 1 diabetes and lower vaccine uptake in participants who had received at least one vaccination through more negative beliefs about the COVID-19 vaccine’s effectiveness (Table 4).

#### 3.4.4. Speed of Vaccine Development Subscale

Female gender and younger age were associated with greater negative attitudes towards speed of vaccine development (Supplementary Table S9). Participants who reported their annual household income to be AUD50K (50,000 Australian dollars) or more, or those who did not disclose their income, reported higher negative scores on the Speed of Vaccine Development Subscale, when compared to participants with an annual household income of less than AUD50K. Conversely, English as a non-dominant language and type 2 diabetes were both associated with lower scores on the speed of vaccine development subscale. Participants unsure of their most recent HbA1c also reported lower scores on the speed of vaccine development than participants who reported their most recent HbA1c as <7% (53 mmol/mol). In the multivariable analysis, age, annual household income of AUD100K to 150K, and English as a non-dominant language remained significant (Supplementary Table S10). There was a partially mediated relationship between younger age and lower vaccine uptake through higher concerns about speed of vaccine development when analyzed with any (one or two) versus no vaccination; this relationship was fully mediated for the group who received two vaccinations versus one or none (Table 4). There was a partially mediated relationship between type 1 diabetes and lower vaccine uptake for participants who had received two vaccinations and a fully mediated relationship when analyzed for participants who had received any vaccinations (one or two doses) through greater concerns about the speed of vaccine development (Table 4).

#### 3.4.5. Side-Effects Subscale

Female gender and younger age were associated with higher negative attitudes towards vaccine side-effects (Supplementary Table S11). Participants who self-reported their diabetes management over the past month as either “poor”, “fair”, “good”, or “very good” reported stronger negative attitudes towards vaccine side-effects compared with those who self-reported “excellent” diabetes management. Higher side-effects scores were also reported by participants who indicated their diabetes impacted on their daily activities either “all of the time” or “most of the time”, compared to those who reported less frequent impact. In the multivariable analysis, age, self-reported diabetes management (“fair”, “good”, and “very good”), and “not at all”/”not very often” impact of diabetes on daily activities remained significant (Supplementary Table S12). A partially mediated relationship between younger age and lower vaccine uptake was seen through higher concerns about vaccine side-effects when analyzed for the group receiving any vaccination (1 or 2), which was fully mediated when analyzed for the group who had received two vaccinations compared with one or none (Table 4). There was a partially mediated relationship between type 1 diabetes and lower vaccine uptake for participants who had received two vaccinations and a fully mediated relationship when analyzed for participants who had received any vaccinations (one or two) through greater concerns about vaccine side-effects.

### 3.5. Impact of Underlying Disease (DIVAS-6)

#### 3.5.1. Response Frequencies

**Figure 1:**
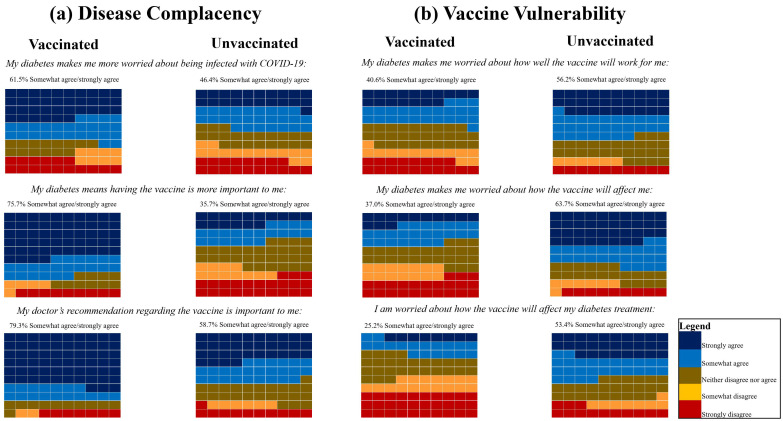
Waffle plots illustrating participants’ response choices for the items of each DIVAS-6 subscale, by vaccination status: (**a**) disease complacency and (**b**) vaccine vulnerability. Each individual colored box represents 1% of responses. Abbreviations: DIVAS-6, Disease Influenced Vaccine Acceptance Scale 6.

Unvaccinated participants reported significantly higher Disease Complacency Subscale scores, indicating greater perceived complacency with COVID-19 infection in the context of diabetes. When compared with vaccinated participants, they were less likely to agree with the following items: (1) “My diabetes makes me worried about being infected with COVID-19” (46.4% vs. 61.5%); (2) “My diabetes means having the vaccine is more important to me” (35.7% vs. 75.7%); and (3) “My doctor’s recommendation regarding the vaccine is important to me” (58.7% vs. 79.3%) (Figure 1a).

Unvaccinated participants also reported significantly higher Vaccine Vulnerability Subscale scores, indicating greater concerns about the COVID-19 vaccine impacting on their diabetes disease course and/or treatment. When compared with vaccinated participants, they were more likely to agree with the following items: (1) “My diabetes makes me worried about how well the vaccine will work for me” (56.2% vs. 40.6%); (2) “My diabetes makes me worried about how the vaccine will affect me” (63.7% vs. 37.0%); and (3) “I am worried about how the vaccine will affect my diabetes treatment” (53.4% vs. 25.2%) (Figure 1b). Response frequencies when vaccinated status was defined as two doses are provided in Supplementary Figure S2.

#### 3.5.2. Disease Complacency Subscale

Lower scores on the Disease Complacency Subscale, indicating greater COVID-19 concerns, were reported by participants with university education, compared to participants with secondary or lower education. Participants who self-reported their diabetes management over the past month as “fair”, “good”, and “very good” also reported a lower subscale score when compared to participants who self-reported “excellent” (Supplementary Table S13). On the multivariable regression, “fair” to “very good” self-reported diabetes management and university education remained significantly associated with lower Disease Complacency Subscale scores (Supplementary Table S14). There was a partially mediated relationship between having type 1 diabetes and lower vaccine uptake though higher disease complacency (Table 4). There was a partially mediated relationship between longer diabetes duration and higher vaccine uptake for participants who had received two vaccinations through lower disease complacency.

#### 3.5.3. Vaccine Vulnerability Subscale

“Poor” and “fair” self-reported diabetes management over the past month were associated with a higher subscale score (Supplementary Table S13), indicating more concerns about the vaccine affecting their diabetes or treatment. Furthermore, participants who reported that their diabetes impacted their daily activities “all of the time” or “most of the time” reported higher Vaccine Vulnerability Subscale scores compared to participants who reported “not at all” or “not very often”. When entering these together in a multivariable analysis, they remained significant (Supplementary Table S15). There was a partially mediated relationship between younger age and lower vaccine uptake through lower disease vulnerability (Table 4).

## 4. Discussion

### 4.1. Main Findings

This study found that COVID-19 vaccine hesitancy and uptake in people with diabetes were influenced by diabetes-specific factors, including concern for the impact of diabetes on vaccine efficacy and, conversely, how the vaccine may interfere with diabetes control. In our study, vaccine uptake was associated with older age and type 2 diabetes, whereas younger age was the only factor predictive of vaccine hesitancy. Our study found that younger age and lower vaccination status were partially mediated by higher vaccine hesitancy, greater vaccine-related negative attitudes and vaccine-effectiveness beliefs, concerns about speed of vaccine development and side-effects, and higher perceived disease-related vaccine vulnerability. The mediation analysis also showed that the relationship between type 1 diabetes and lower vaccination was strengthened by higher vaccine hesitancy, greater negative beliefs about the COVID-19 vaccine’s effectiveness, concerns about speed of development and side-effects from the vaccine, and greater disease complacency with relation to COVID-19. There is limited research on COVID-19 vaccine hesitancy in people with diabetes, and to our knowledge, this is the first Australian study.

In our study, 82.7% of participants had received at least one dose of the vaccine, which was slightly higher than the general population at the time (79.9%) [23]. This may be partially explained by the prioritization of people with chronic diseases in the Australian vaccine rollout [6]. Similar international studies of people with diabetes report much lower vaccination rates, i.e., between 5.1% and 34.7% [9,10,11,24]. While the definition of “vaccinated” generally included those with one or more doses of the COVID-19 vaccine, similar to our study, there are several possible factors underlying these wide variations and overall low vaccination rates compared to our study. Our study was conducted during the global vaccination campaign and five months after people with diabetes were eligible in Australia, when COVID-19 vaccines were easily accessible. In an Italian study [9], the survey was conducted in January 2021, when there were significant delays in the vaccine rollout due to limited supply, and 73.1% of respondents were female, which is a factor associated with vaccine hesitancy [9,10,16,17,25]. A Chinese survey conducted in 2021 [10] included only inpatients as participants, limiting generalizability to the broader population of people with diabetes, and 59.6% of the sample had less than high school education, which is a known risk factor for vaccine hesitancy [9,10,16,17,25]. Vaccination rates were similarly low in a Saudi Arabian study [11]; though, of note, more than two-thirds of participants had type 1 diabetes, which may be associated with lower vaccine uptake. Indeed, in the current study, participants with type 1 diabetes were more likely to be unvaccinated against COVID-19 compared with people with type 2 diabetes, independent of age. We also found that higher vaccine hesitancy, lower collective importance, concerns about speed of vaccine side-effects and speed of development, and higher disease complacency mediated the relationship between type 1 diabetes and lower vaccination.

There is limited literature comparing vaccine uptake between diabetes types. In an Italian survey [15], people with type 2 diabetes reported higher vaccine hesitancy, and in a Saudi Arabian survey [11], there was no significant difference in vaccine hesitancy between diabetes types. Further research is needed to understand and address vaccine hesitancy in people with type 1 diabetes, as they are at equal if not higher risk of severe disease, complications, and mortality from COVID-19 compared with those with type 2 diabetes [4,26,27]. Clinician recommendations and broader public health messaging should focus on people with type 1 diabetes as a high-risk cohort.

### 4.2. Vaccine Vulnerability

In our study, a significant proportion of participants (53.4% unvaccinated participants and 25.2% vaccinated participants) reported concern about the vaccine affecting diabetes treatment. Two groups who reported greater vaccine vulnerability were those who described a greater impact of diabetes on their daily activities (“all of the time” or “most of the time”) and those with the worst self-reported diabetes control (“poor” or “fair”). It is possible that those who experience a greater diabetes-related burden on daily activities, such as more complex insulin regimens with numerous injections, a greater pill burden, and more frequent blood glucose monitoring, may feel especially cautious towards side-effects or diabetes destabilization, which may further add to their self-management demands. Similarly, participants who report worse diabetes control may be having difficulty achieving glycemic targets and may avoid the COVID-19 vaccine due to fear that it further worsens diabetes control, a relationship has been reported previously [28]. Similar reasons for vaccine hesitancy appear to be shared by other high-risk chronic disease groups including end-stage renal failure, rheumatological disease, inflammatory disease, and HIV [18,29,30,31,32,33]. In an Australian study of people with rheumatological diseases, vaccine hesitancy correlated with concerns regarding side-effects and vaccine-associated flare of rheumatological diseases [30]. In dialysis patients in France and Italy, vaccine hesitancy was low overall at 11.3% and was associated with concerns about side-effects and vaccine efficacy [29]. Overall, this shows that in addition to common concerns shared with the general population, individuals with chronic diseases like diabetes also have significant concerns around disease destabilization.

### 4.3. People from Culturally and Linguistically Diverse (CALD) Backgrounds

In our study, a high proportion of people reported English as a non-dominant language (19.6%), reflecting the diverse population attending the participating practices. Participants who speak English as a non-dominant language indicated greater perceived collective importance of the COVID-19 vaccination and less concern regarding the rapid speed of vaccine development compared to those who speak English as their dominant language. Cultural differences are likely to play a role in vaccine attitudes. Cultural orientation exists along a spectrum of individualism to collectivism and is highly relevant to vaccine beliefs [34]. In individualistic cultures (most Western countries), people prioritize independence and self-sufficiency, whereas in collectivist cultures (most Asian countries), groups are of primary importance, and individuals are seen as secondary [35]. Participants in a cross-national study who identified more with collectivism were more likely to accept COVID-19 vaccines [34]. In keeping with these findings, our results showed that participants who speak English as a non-dominant language were more likely to consider COVID-19 vaccination to be of collective importance for the community. However, in our cohort, there was ultimately no significant difference in vaccine uptake between those who speak or do not speak English as a dominant language, likely due to multiple clinical and non-clinical factors interacting with cultural factors to contribute to vaccine behaviors.

### 4.4. Study Implications

In our study, the majority of people (79.3% of vaccinated participants and 58.7% of unvaccinated participants) agreed that their clinician’s recommendation for the COVID-19 vaccination is important to them, suggesting that targeted clinician-led discussions are powerful in shaping vaccination attitudes. Such discussions should be included in routine diabetes consultations. In an Australian study of people with rheumatic diseases, 54.4% of vaccine-hesitant participants were more likely to accept vaccination if recommended by their specialist [30]. Given that people with diabetes share similar concerns with people with other chronic diseases, it follows that specific COVID-19 vaccine recommendations by diabetes clinicians can increase vaccine acceptance in those who are hesitant [19]. The DIVAS-6 can be utilized during consultations to facilitate targeted education. Those scoring highly on the Disease Complacency Subscale may feel they have little to gain from undergoing vaccination, and this should prompt discussion around the increased risk of severe COVID-19 disease. This is particularly relevant for people with a shorter disease duration, where there was a mediating relationship with reduced vaccination when disease complacency was higher. Similarly, a high score on the Vaccine Vulnerability Subscale should lead to discussion around the safety of the vaccine. This approach is most essential for younger patients and those with type 1 diabetes, two groups which are more likely to be unvaccinated.

Furthermore, despite evidence for the safety and efficacy of COVID-19 vaccination in people with diabetes [36,37,38], several participants expressed a fear of side-effects, particularly younger people; this was one of the most common drivers of vaccine hesitancy in other studies of people with diabetes [9,10,24]. Our study found that younger age and vaccination status were mediated through higher concerns about side-effects and vaccine speed of development. This reveals an information gap which needs to be addressed, especially with the need for ongoing booster doses. Currently, recommendations around COVID-19 vaccination are available from diabetes organizations in Australia and internationally [7,37,38]. To help close the information gap, these recommendations should be more widely communicated as public health messages, including materials targeted to people from CALD backgrounds.

### 4.5. Strengths and Limitations

The strengths of this study include the large, diverse cohort of people with diabetes from metropolitan and regional/rural Australia. This study used a disease-specific scale, in addition to other validated scales, to explore the underlying drivers of vaccine hesitancy in relation to diabetes and other factors. Our mediation analysis showed the interlink between demographics (age) and disease-related factors (type 1 or 2; diagnosis duration; treatment status) and vaccination status. A significant limitation was a low response rate (16.5% of eligible participants), which may be partially attributed to pandemic-related survey fatigue [39]. This may limit the generalizability of the results, as selection bias cannot be excluded. Additionally, there is potential for recall and misclassification bias given the survey-based study design. We did not evaluate broader social and political factors which may influence vaccine hesitancy alongside the clinical factors assessed. As COVID-19 incidence, vaccine availability, and community transmission rates varied between Australia and many other countries during the first year of the pandemic, caution is required with the direct applicability of our findings to other regions of the world [40].

## 5. Conclusions

In this study of a large, diverse cohort of people with diabetes in Australia, high rates of vaccination were reported, although a significant proportion held specific diabetes-related concerns which contributed to vaccine hesitancy. With the emergence of new viral variants and ongoing need for booster doses, it is important to understand and address these concerns in people with diabetes, who are particularly vulnerable to COVID-19 disease and complications. This study sheds light on important patient-related and diabetes-related risk factors for vaccine hesitancy, enabling clinicians to identify and support high-risk subgroups within the diabetes population, particularly young people and those with type 1 diabetes.

## Figures and Tables

**Table 1:** Participant characteristics.

	All Participants *n* = 842 *n*, (%)	Vaccinated *n* = 696 (82.7%) *n*, (%)	Not Vaccinated *n* = 146 (17.3%) *n*, (%)
Male	457 (54.3)	383 (83.8)	74 (16.2)
Female *	378 (44.9)	307 (81.2)	71 (18.8)
**Age: median**	58 (IQR 19)	59 (IQR 18)	53 (IQR 22)
**Age (years)**			
18–49	251 (29.8)	188 (74.9)	63 (25.1)
50–69	455 (54.0)	384 (84.4)	71 (15.6)
≥70	136 (16.2)	124 (91.2)	12 (8.8)
**Highest level of education** †			
No formal education/primary school	37 (4.4)	27 (73.0)	10 (27.0)
Secondary school	319 (37.9)	268 (84.0)	51 (16.0)
Vocational/trade	231 (27.4)	189 (81.8)	42 (18.2)
University	253 (30.0)	212 (83.8)	41 (16.2)
**Annual household income (AUD)**			
<50 K	323 (38.4)	283 (87.6)	40 (12.4)
50–100 K	217 (25.8)	175 (80.5)	42 (19.4)
100–150 K	92 (10.9)	70 (76.1)	22 (23.9)
>150 K	52 (6.2)	42 (80.8)	10 (19.2)
Prefer not to say	158 (18.8)	126 (79.7)	32 (20.3)
**Aboriginal/Torres Strait Islander** ‡			
Yes	25 (3.0)	21 (84.0)	4 (16.0)
**English as first language**			
Yes	677 (80.4)	554 (81.8)	123 (18.2)
No	165 (19.6)	142 (86.1)	23 (13.9)
**Location**			
Metropolitan	605 (71.9)	497 (82.1)	108 (17.9)
Regional	237 (28.1)	199 (84.0)	38 (16.0)
**Diabetes Type**			
Type 1	252 (29.9)	189 (75.0)	63 (25.0)
Type 2	557 (66.2)	482 (86.5)	75 (13.5)
Other/don’t know	33 (3.9)	25 (75.8)	8 (24.2)
**Time since diagnosis**			
<1 year	37 (4.4)	30 (81.1)	7 (18.9)
1–5 years	106 (12.6)	82 (77.4)	24 (22.6)
5.1–10 years	174 (20.7)	142 (81.6)	32 (18.4)
>10 years	525 (62.4)	442 (84.2)	83 (15.8)
**Most recent HbA1c within the past year**			
<7%	139 (16.6)	114 (82.0)	25 (18.0)
7–8.5%	334 (39.9)	284 (85.0)	50 (15.0)
8.6–10%	148 (17.7)	123 (83.1)	25 (16.9)
>10%	67 (8.0)	56 (83.6)	11 (16.4)
Don’t know	150 (17.9)	117 (78.0)	33 (22.0)
**Current diabetes treatment**			
Insulin	279 (33.1)	211 (75.6)	68 (24.4)
Tablets	173 (20.6)	145 (83.8)	28 (16.2)
Injectables (not insulin)	11 (1.3)	8 (72.7)	3 (27.3)
Diet only	15 (1.8)	13 (86.7)	2 (13.3)
Combination of treatments/other	364 (43.2)	319 (87.6)	45 (12.4)
**Management of diabetes in the past month:**			
Poor	46 (5.5)	34 (73.9)	12 (26.1)
Fair	169 (20.1)	135 (79.9)	34 (20.1)
Good	302 (35.9)	252 (83.4)	50 (16.6)
Very good	237 (28.2)	198 (83.5)	39 (16.5)
Excellent	87 (10.3)	76 (87.4)	11 (12.6)
**Diabetes affected daily activities in last 4 weeks**			
All the time	52 (6.2)	40 (76.9)	12 (23.1)
Most of the time	92 (10.9)	76 (82.6)	16 (17.4)
Some of the time	222 (26.4)	182 (82.0)	40 (18.0)
Not very often	229 (27.2)	191 (83.4)	38 (16.6)
Not at all	246 (29.3)	206 (83.7)	40 (16.3)

**Notes:** * There was also “non-binary/other” (*n* = 7, 0.8%). † There was also “other” (*n* = 2, 0.2%). ‡ There was also “prefer not to say” (*n* = 12, 1.4%). Abbreviations: IQR, interquartile range; AUD, Australian dollars; K, 1000; HbA1c, glycated hemoglobin.

**Table 2:** Logistic regression analysis predicting vaccinated status with sociodemographic and clinical characteristics.

Category (Reference, *n*)	B (SE)	OR (95% CI)	*p*-Value
**Age (*n* = 842)**	0.03 (0.006)	1.03 (1.02–1.04)	<0.001
**Diabetes type (type 1, *n* = 809)**			
Type 2	0.87 (0.20)	2.39 (1.61–3.53)	<0.001
**Current diabetes treatment (yes, *n* = 842)**			
No	−0.12 (0.65)	0.88 (0.25–3.15)	0.85

**Notes:** Regression analyses were controlled for time since study commencement. Diabetes type “don’t know/other” was excluded for comparisons between Type 1 and Type 2 diabetes. Abbreviations: B (SE), unstandardized coefficient (standard error); OR (95% CI), odds ratio (95% confidence interval).

**Table 3:** Linear regression analysis predicting the Oxford COVID-19 Vaccine Hesitancy Scale summary score with sociodemographic and clinical characteristics.

	Step 1	Step 2			
Category (Reference, *n*)	Adj. R^2^	Adj. R^2^	Δ Adj. R^2^	B (SE)	*p*-Value
**Age (*n* = 780)**	0.002	0.015	0.013	−0.05 (0.01)	0.001
**Current diabetes treatment (yes, *n* = 780)**	0.002	0.00	−0.002		
No				0.25 (1.50)	0.87
**Diabetes management over the past month (excellent, *n* = 779)**	0.002	0.003	0.001		
Poor				1.89 (1.16)	0.10
Fair				1.46 (0.79)	0.07
Good				0.88 (0.73)	0.23
Very good				1.30 (0.76)	0.09

**Notes:** Regression analyses were controlled for time since study commencement at step 1. Abbreviations: Adj. R^2^, Adjusted R^2^; B (SE), unstandardized coefficient (standard error).

**Table 4:** Results of the mediation analysis, where the scale and subscale scores are the mediators, between sociodemographic and disease variables (independent variables) and vaccinated status (dependent variable).

Mediator	Dependent Variable	*a*	*b*	*c’*	Indirect Effect	Bootstrapped 95% CIs for IE
**Independent Variable: Age**						
OCVHS score	Vaccinated status (≥1 dose)	**−0.05**	**0.27**	**−0.03**	**−0.01**	**−0.02, −0.005 ^**
	Vaccinated status (2 doses)	**−0.05**	**0.27**	−0.008	**−0.01**	**−0.02, −0.005 ^^**
OCVCCS—Summary score	Vaccinated status (≥1 dose)	**−0.09**	**0.15**	**−0.02**	**−0.01**	**−0.02, −0.006 ^**
	Vaccinated status (2 doses)	**−0.09**	**0.10**	−0.008	**−0.009**	**−0.02, −0.004 ^^**
OCVCCS—Beliefs about COVID-19 Vaccine score	Vaccinated status (≥1 dose)	**−0.01**	**0.59**	**−0.03**	**−0.007**	**−0.01, −0.0002 ^**
	Vaccinated status (2 doses)	**−0.01**	**0.35**	**−0.02**	**−0.004**	**−0.008, −0.0002 ^**
OCVCCS—Collective Importance score	Vaccinated status (≥1 dose)	−0.02	**0.44**	**−0.03**	−0.007	−0.01, 0.0007
	Vaccinated status (2 doses)	−0.02	**0.26**	**−0.01**	−0.004	−0.009, 0.0004
OCVCCS—Speed of Vaccine Development score	Vaccinated status (≥1 dose)	**−0.034**	**0.51**	**−0.02**	**−0.02**	**−0.03, −0.01 ^**
	Vaccinated status (2 doses)	**−0.034**	**0.26**	−0.008	**−0.009**	**−0.014, −0.005 ^^**
OCVCCS—Side-Effects score	Vaccinated status (≥1 dose)	**−0.03**	**0.48**	**−0.03**	**−0.02**	**−0.02, −0.01**
	Vaccinated status (2 doses)	**−0.03**	**0.36**	−0.008	**−0.01**	**−0.02, −0.008 ^^**
DIVAS-6—Disease Complacency score	Vaccinated status (≥1 dose)	0.01	**0.22**	**−0.04**	0.002	−0.001, 0.006
	Vaccinated status (2 doses)	0.01	**0.13**	**−0.02**	0.001	−0.0005, 0.004
DIVAS-6—Vaccine Vulnerability score	Vaccinated status (≥1 dose)	−0.02	**0.19**	**−0.03**	−0.003	−0.008, 0.0002
	Vaccinated status (2 doses)	−0.02	**0.13**	**−0.02**	−0.002	−0.005, 0.0001
**Independent Variable: Diabetes Type**						
OCVHS score	Vaccinated status (≥1 dose)	**−1.44**	**0.27**	**−0.63**	**−0.39**	**−0.70, −0.13 ^**
OCVCCS—Summary score	Vaccinated status (≥1 dose)	**−1.64**	**0.17**	−0.51	−0.27	−0.60, 0.008
OCVCCS—Beliefs about COVID-19 Vaccine score	Vaccinated status (≥1 dose)	**−0.52**	**0.62**	**−0.68**	**−0.32**	**−0.62, −0.08 ^**
OCVCCS—Collective Importance score	Vaccinated status (≥1 dose)	**−0.69**	**0.46**	**−0.69**	**−0.32**	**−0.60, −0.08 ^**
OCVCCS—Speed of Vaccine Development score	Vaccinated status (≥1 dose)	**−0.73**	**0.54**	**−0.59**	**−0.39**	**−0.69, −0.14 ^**
OCVCCS—Side-Effects score	Vaccinated status (≥1 dose)	**−0.41**	**0.53**	**−0.62**	**−0.22**	**−0.44, −0.02 ^**
DIVAS-6—Disease Complacency score	Vaccinated status (≥1 dose)	**0.68**	**0.23**	**−1.19**	**0.16**	**0.04, 0.28 ^**
DIVAS-6—Vaccine Vulnerability score	Vaccinated status (≥1 dose)	−0.17	**0.20**	**−0.81**	−0.03	−0.17, 0.09
**Independent Variable: Time Since Diabetes Diagnosis**						
OCVHS score	Vaccinated status (2 doses)	0.15	**0.28**	**−0.32**	0.04	−0.09, 0.17
OCVCCS—Summary score	Vaccinated status (2 doses)	−0.20	**0.10**	**−0.25**	−0.02	−0.11, 0.07
OCVCCS—Beliefs about COVID-19 Vaccine score	Vaccinated status (2 doses)	−0.06	**0.36**	**−0.22**	−0.02	−0.09, 0.04
OCVCCS—Collective Importance score	Vaccinated status (2 doses)	0.20	**0.28**	**−0.29**	0.05	−0.02, 0.13
OCVCCS—Speed of Vaccine Development score	Vaccinated status (2 doses)	−0.04	**0.27**	**−0.20**	−0.11	−0.08, 0.05
OCVCCS—Side-Effects score	Vaccinated status (2 doses)	−0.01	**0.37**	**−0.29**	−0.005	−0.08, 0.06
DIVAS-6—Disease Complacency score	Vaccinated status (2 doses)	**−0.30**	**0.12**	**−0.21**	**−0.03**	**−0.07, −0.003 ^**
DIVAS-6—Vaccine Vulnerability score	Vaccinated status (2 doses)	0.15	**0.14**	−0.29	0.02	−0.02, 0.07
**Independent Variable: Diabetes Treatment**						
OCVHS score	Vaccinated status (≥1 dose)	0.25	**0.28**	0.65	0.07	−0.85, 1.30
	Vaccinated status (2 doses)	0.25	**0.27**	−0.10	0.07	−0.85, 1.27
OCVCCS—Summary score	Vaccinated status (≥1 dose)	−1.12	**0.15**	0.37	−0.17	−0.76, 0.43
	Vaccinated status (2 doses)	−1.12	**0.10**	−0.44	−0.11	0.49, 0.29
OCVCCS—Beliefs about COVID-19 Vaccine score	Vaccinated status (≥1 dose)	0.24	**0.61**	0.63	0.15	−0.81, 1.44
	Vaccinated status (2 doses)	0.24	**0.36**	−0.25	0.08	−0.49, 0.83
OCVCCS—Collective Importance score	Vaccinated status (≥1 dose)	−1.26	**0.44**	0.30	**−0.55**	**−1.01, −0.14 ^^**
	Vaccinated status (2 doses)	−1.26	**0.26**	−0.19	**−0.33**	**−0.61, −0.08 ^^**
OCVCCS—Speed of Vaccine Development score	Vaccinated status (≥1 dose)	0.90	**0.53**	−0.007	0.47	−0.39, 1.50
	Vaccinated status (2 doses)	0.90	**0.27**	−0.35	0.25	−0.20, 0.76
OCVCCS—Side-Effects score	Vaccinated status (≥1 dose)	0.13	**0.50**	0.02	0.07	−0.56, 0.77
	Vaccinated status (2 doses)	0.13	**0.37**	−0.44	0.05	−0.41, 0.56
DIVAS-6—Disease Complacency score	Vaccinated status (≥1 dose)	−0.04	**0.20**	0.52	−0.008	−0.36, 0.39
	Vaccinated status (2 doses)	−0.04	**0.12**	0.05	−0.005	−0.23, 0.24
DIVAS-6—Vaccine Vulnerability score	Vaccinated status (≥1 dose)	−0.93	**0.20**	0.64	−0.18	−0.62, 0.19
	Vaccinated status (2 doses)	−0.93	**0.13**	0.14	−0.12	−0.41, 0.13

**Notes:** Bolded values indicates effects that are significant at a level of 0.05 or 95% confidence interval (CI), as determined using bootstrapping methods recommended by A.F. Hayes [21]. ^ Partial mediation; ^^ full mediation. Abbreviations: *a*, regression coefficient of the sociodemographic or disease variable on the scale or subscale score; *b*, regression coefficient of the scale or subscale score on vaccinated status; *c’*, regression coefficient that estimates direct effect of sociodemographic or disease variable on vaccinated status; IE; indirect effect; 95% CIs; 95% confidence intervals; OCVHS; Oxford COVID-19 Vaccine Hesitancy Scale; OCVCCS; Oxford COVID-19 Vaccine Confidence and Complacency Scale; DIVAS-6; Disease Influenced Vaccine Acceptance Scale Six.

## Data Availability

The data presented in this study are available upon reasonable request from the corresponding author.

## Supplementary Materials

The following supporting information can be downloaded at https://www.mdpi.com/article/10.3390/vaccines12060662/s1, Figure S1: Survey study timeline for each participating health service site, relative to state-wide strict lockdowns for COVID-19 and COVID-19 vaccine milestones. Figure S2: Waffle plots illustrating participants’ response choices for the items of each DIVAS-6 subscale by vaccination status (defined as two doses): (a) disease complacency and (b) vaccine vulnerability. Table S1: Survey items. Table S2: Participant characteristics (vaccinated status is defined as two doses). Table S3: Hierarchical multivariable logistic regression analysis predicting vaccinated status with sociodemographic and clinical characteristics (*n* = 809). Table S4: Logistic regression analysis predicting vaccinated status (defined as two doses) with sociodemographic and clinical characteristics. Table S5: Hierarchical multivariable logistic regression analysis predicting vaccinated status (defined as two doses) with sociodemographic and clinical characteristics (*n* = 809). Table S6: Linear regression analysis predicting the Oxford COVID-19 Vaccine Confidence and Complacency Scale—Summary Scale score and the Collective Importance and Beliefs about COVID-19 Vaccine Subscale scores with sociodemographic and clinical characteristics. Table S7: Hierarchical multivariable linear regression analysis of the Oxford COVID-19 Vaccine Confidence and Complacency Scale score (*n* = 467). Table S8: Hierarchical multivariable linear regression analysis of the Oxford COVID-19 Vaccine Confidence and Complacency—Collective Importance score (*n* = 679). Table S9: Linear regression analysis predicting the Oxford COVID-19 Vaccine Confidence and Complacency Scale—Speed of Vaccine Development Subscale score with sociodemographic and clinical characteristics. Table S10: Hierarchical multivariable linear regression analysis of the Oxford COVID-19 Vaccine Confidence and Complacency Scale—Speed of Vaccine Development (*n* = 651). Table S11: Linear regression analysis predicting the Oxford COVID-19 Vaccine Confidence and Complacency Scale—Side-Effects Subscale score with sociodemographic and clinical characteristics. Table S12: Hierarchical multivariable linear regression analysis of the Oxford COVID-19 Vaccine Confidence and Complacency Scale—Side-Effects Subscale score (*n* = 694). Table S13: Linear regression analysis predicting the Disease Influenced Vaccine Acceptance Scale Six—Disease Complacency and Vaccine Vulnerability Subscale scores with sociodemographic and clinical characteristics. Table S14: Multivariable linear regression analysis of the Disease Influenced Vaccine Acceptance Scale Six—Disease Complacency score (*n* = 770). Table S15: Multivariable linear regression analysis predicting the Disease Influenced Vaccine Acceptance Scale Six—Vaccine Vulnerability Subscale score with sociodemographic and clinical characteristics (*n* = 724).
