# Supplementary material for: COVID-19 Vaccine Acceptance, Hesitancy, and Uptake in People with Diabetes in Australia
Source: Vaccines (Basel). 2024 Jun 16;12(6):662. doi: 10.3390/vaccines12060662 (PMC11209119; doi:10.3390/vaccines12060662)
Supplement: Supplementary file 1 [file vaccines-12-00662-s001.zip › vaccines-2997941-supplementary.pdf]

Supplementary materials

These supplementary materials have been provided by the authors to give readers additional information about their work.

Supplement to: COVID-19 vaccine acceptance, hesitancy, and uptake in people with diabetes in Australia.

List of the DIABetes patients' perspectives on coronavirus VACCination Survey (DIABVACCS) investigators.

Monash Health, Victoria: Dr. Nathan Bain, Dr. Daphne Day, Prof. Barbora de Courten, Dr. Lisa Grech, Dr. Amelia McCartney, Dr. Mike Nguyen, Prof. Eva Segelov, Dr Holly Wang, Dr. Kate Webber, A/Prof. Jennifer Wong.

Bendigo Health, Victoria: Dr. Frank Gao, Dr. Amy Harding, A/Prof. Mark Savage.

Sunshine Coast Hospital and Health Service, Queensland: Dr. Brett Sillars.

Dr David Hoffman, New South Wales: Dr. David Hoffman.

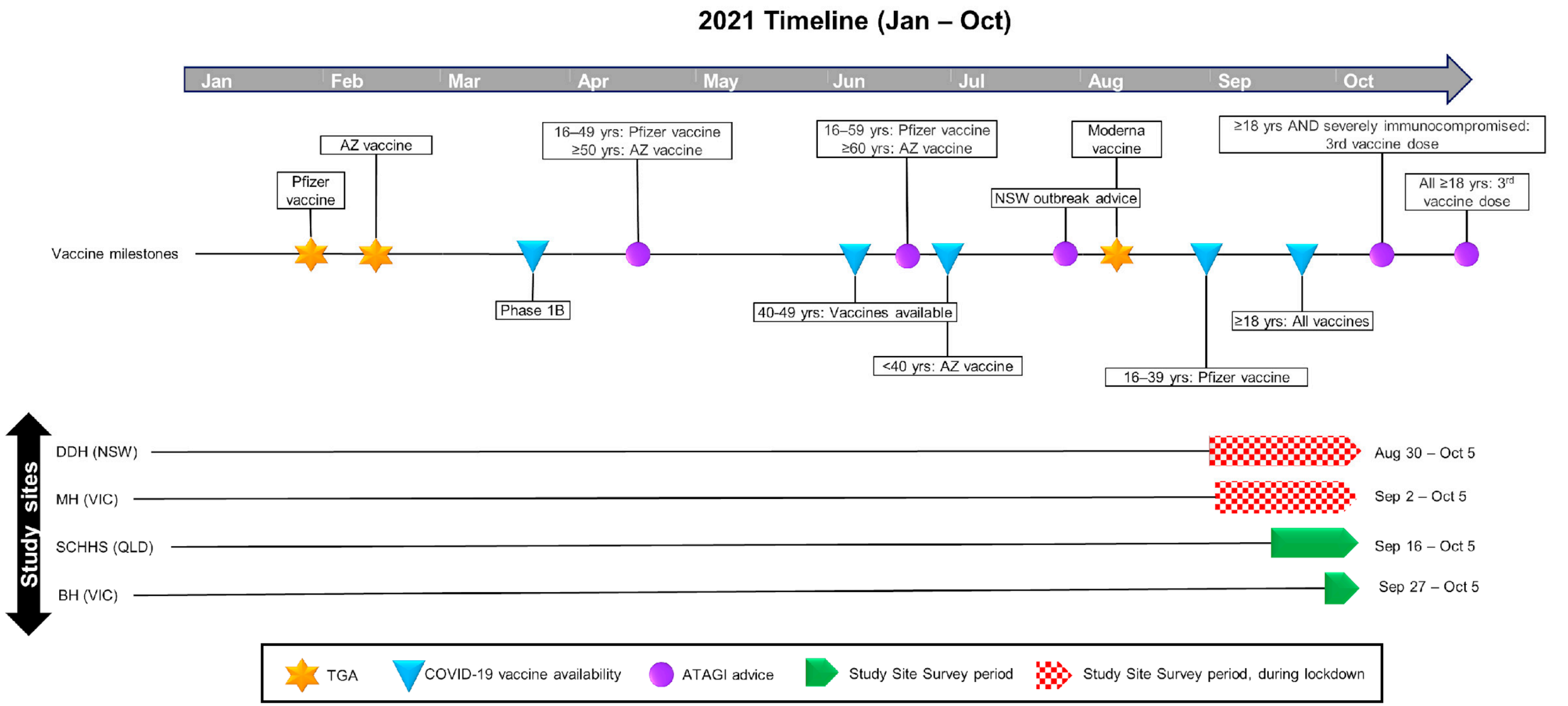

Figure S1. Survey study timeline for each participating health service site, relative to state-wide strict lockdowns for COVID-19 and COVID-19 vaccine milestones. Abbreviations: AZ, Astra-Zeneca; Years, yrs; NSW, New South Wales; DDH, Dr David Hoffman; MH, Monash Health; VIC, Victoria; SCHHS, Sunshine Coast Hospital and Health Service; QLD, Queensland; Bendigo Health, BH; TGA, Therapeutic Goods Administration – COVID-19 Vaccine Provisional Registration; ATAGI, Australian Technical Advisory Group on Immunisation. People with diabetes were eligible for COVID-19 vaccination from the commencement of the Australian Government Rollout Phase 1B.

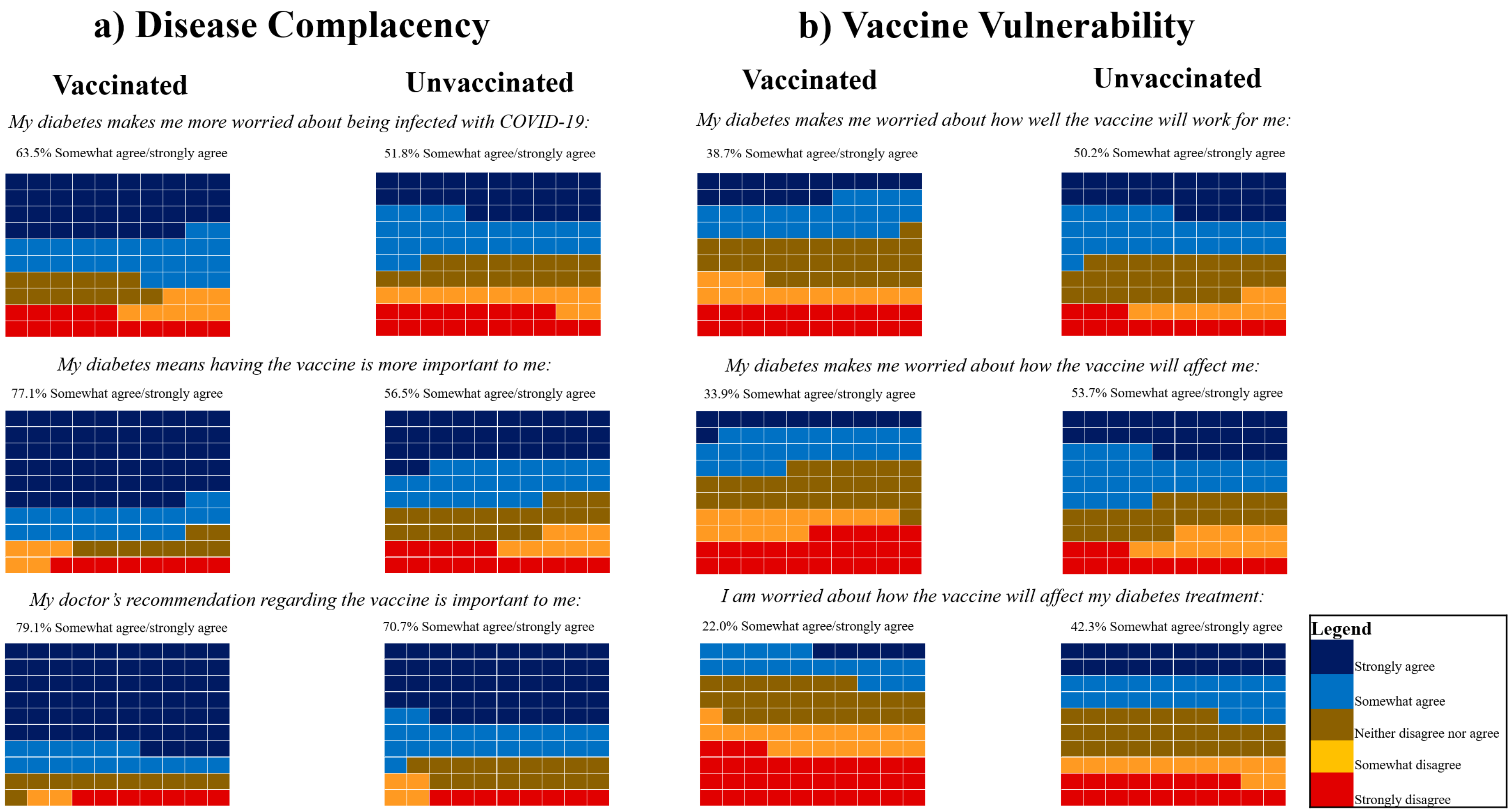

Figure S2. Waffle plots illustrating participants' response choices for the items of each DIVAS-6 subscale, by vaccination status (defined as two doses). a) Disease Complacency; b) Vaccine Vulnerability. Each individual-coloured box represents 1% of responses. Abbreviations: DIVAS-6, Disease Influenced Vaccine Acceptance Scale-6.

Table S1. Survey items

**Screening items**		
	Are you 18 years or older?	Yes<br>No (Terminate if No)
	Have you got diabetes?	Yes<br>No (Terminate if No)
	Are you a [participating site] patient?	Yes<br>No (Terminate if No)
**Vaccination status**		
	Have you already received a COVID-19 vaccine?	Yes, 1 dose only<br>Yes, 2 doses<br>No
**Oxford COVID-19 Vaccine Hesitancy Scale**<br>Instructions: We would like to know your feelings and thoughts about the COVID-19 vaccine. Note: If you have already been vaccinated against COVID-19, please complete these questions in relation to a future COVID-19 vaccine dose/booster.		
	Would you take a COVID-19 vaccine if offered?	Definitely/have taken<br>Probably<br>I may or may not<br>Probably not<br>Definitely not<br>Don't know
	When a COVID-19 vaccine is available:	I will want to get it as soon as possible<br>I will take it when offered<br>I'm not sure what I will do<br>I will put off (delay) getting it<br>I will refuse to get it<br>Don't know
	I would describe my attitude towards receiving a COVID-19 vaccine as:	Very keen<br>Pretty positive<br>Neutral<br>Quite uneasy<br>Against it

	Don't know
If a COVID-19 vaccine was available in my local area, I would:	Get it as soon as possible<br>Get it when I have time<br>Delay getting it<br>Avoid getting it for as long as possible<br>Never get it<br>Don't know
If my family or friends were thinking of getting a COVID-19 vaccination, I would:	Strongly encourage them<br>Encourage them<br>Not say anything to them about it<br>Ask them to delay getting the vaccination<br>Suggest that they do not get the vaccination<br>Don't know
I would describe myself as:	Eager to get a COVID-19 vaccine<br>Willing to get the COVID-19 vaccine<br>Not bothered about getting the COVID-19 vaccine<br>Unwilling to get the COVID-19 vaccine<br>Anti-vaccination for COVID-19<br>Don't know
Taking a COVID-19 vaccination is:	Really important<br>Important<br>Neither important nor unimportant<br>Unimportant<br>Really unimportant<br>Don't know

Oxford COVID-19 Vaccine Confidence and Complacency Scale

Do you think you will be infected with COVID-19 over the next 12 months?	Definitely<br>Probably<br>Possibly<br>Probably not<br>Definitely not<br>Don't know

I think the COVID-19 vaccine is likely to:	Work for almost everyone<br>Work for most people<br>I am unsure how many people it will work for<br>Not work for most people<br>Not work for anyone<br>Don’t know
I think the COVID-19 vaccine is likely to:	Definitely work for me<br>Probably work for me<br>May or may not work for me<br>Probably not work for me<br>Definitely not work for me<br>Don’t know
I think if I get the COVID-19 vaccine it will be:	Really helpful for the community around me<br>Helpful for the community around me<br>Neither helpful nor unhelpful for the community around me<br>Unhelpful for the community around me<br>Really unhelpful for the community around me<br>Don’t know
I think if individuals like me get the COVID-19 vaccine it will:	Save a large number of lives<br>Save some lives<br>Have no impact<br>Lead to more deaths<br>Lead to a large number of deaths<br>Don’t know
I think the speed of developing and testing the vaccine means it will be:	Really good<br>Good<br>Will not affect how good or bad it is<br>Bad<br>Really bad<br>Don’t know
I think the speed of developing and testing the vaccine means it will be:	Really safe<br>Safe<br>It will not affect how safe it is

	Unsafe<br>Really unsafe<br>Don’t know
I think if many people do not get the vaccine this:	Will be dangerous<br>May be dangerous<br>Will have no consequences at all<br>May be good<br>Will be good<br>Don’t know
I expect that receiving the vaccine will be:	Hardly noticeable<br>A little unpleasant<br>Moderately unpleasant<br>Painful<br>Extremely painful<br>Don’t know
I think the side-effects for people of getting the COVID-19 vaccine will be:	None<br>Mild<br>Moderate<br>Significant<br>Life-threatening<br>Don’t know
I think the COVID-19 vaccine will:	Greatly strengthen my immune system<br>Strengthen my immune system<br>It will neither strengthen nor weaken my immune system<br>Weaken my immune system<br>Greatly weaken my immune system<br>Don’t know
I think taking the COVID-19 vaccine:	Will give me complete freedom to get on with life just as before<br>Will give me greater freedom<br>Will have no effect on my freedom<br>Will restrict my freedom<br>Will completely restrict my freedom to get on with life

	Don't know
I think getting the vaccine is a sign of:	Great personal strength<br>Personal strength<br>Not a sign of personal strength or weakness<br>Personal weakness<br>Great personal weakness<br>Don't know
Taking a new COVID-19 vaccine will make me feel like a guinea pig:	Do not agree<br>Agree a little<br>Agree moderately<br>Agree a lot<br>Completely agree<br>Don't know

Disease Influenced Vaccine Acceptance Scale-Six

Instructions: We would like to know about how your diabetes may be related to your feelings and thoughts about the COVID-19 vaccine. For each of the following statements, please tap/click the one choice that best represents how strongly you agree or disagree with it. There are 6 choices to choose from for each statement.

My history of diabetes makes me more worried about being infected with COVID-19:	Strongly agree<br>Somewhat agree<br>Neither disagree nor agree<br>Somewhat disagree<br>Strongly disagree<br>Don't know
My history of diabetes means having the vaccine is more important to me:	Strongly agree<br>Somewhat agree<br>Neither disagree nor agree<br>Somewhat disagree<br>Strongly disagree<br>Don't know
My doctor's recommendation regarding the vaccine is important to me:	Strongly agree<br>Somewhat agree<br>Neither disagree nor agree<br>Somewhat disagree<br>Strongly disagree

	Don't know
My history of diabetes makes me worried about how well the vaccine will work for me:	Strongly disagree<br>Somewhat disagree<br>Neither disagree nor agree<br>Somewhat agree<br>Strongly agree<br>Don't know
My history of diabetes makes me worried about how the vaccine will affect me:	Strongly disagree<br>Somewhat disagree<br>Neither disagree nor agree<br>Somewhat agree<br>Strongly agree<br>Don't know
I am worried about how the vaccine will affect my diabetes treatment:	Strongly disagree<br>Somewhat disagree<br>Neither disagree nor agree<br>Somewhat agree<br>Strongly agree<br>Don't know
**Clinical**	
What type of diabetes do you have?	Type 1<br>Type 2<br>Other (please specify): ______________<br>Don't know
How long have you had diabetes?	Less than 1 year<br>1 to 5 years<br>5.1 to 10 years<br>More than 10 years
My most recent HbA1c (within the past year) was:	Less than 7%<br>7% to 8.5%<br>8.6% to 10%<br>More than 10%

	Don't know
My current treatment for my diabetes is/are (select all that apply):	Insulin<br>Tablets<br>Diet only<br>Injectables (not insulin)<br>Other:<br>________________________
In the past month, would you say that your management of diabetes was:	Excellent<br>Very good<br>Good<br>Fair<br>Poor
In the last four weeks, how much did your diabetes affect your daily activities?	All the time<br>Most of the time<br>Some of the time<br>Not very often<br>Not at all
**Socio-demographics**	
What is your gender?	Male<br>Female<br>Non-binary / third gender<br>Prefer not to say
What is your age?	[Text entry]:<br>________________________
What is your highest educational level (completed)?	No formal education<br>Primary education<br>Secondary education<br>Vocational/trade qualification<br>University education or higher degree<br>Other (please specify): ________________

What is your annual household income (including everyone who lives in your home)?	Less than $50,000<br>$50,001 to $100,000<br>$100,001 to $150,000<br>More than $150,000<br>Prefer not to say
Do you identify as Aboriginal and/or Torres Strait Islander?	Yes<br>No<br>Prefer not to say
Is English your first language?	Yes<br>No
Please include any comments about your feelings and thoughts about your diabetes and COVID-19 vaccination that you would like to share. If you have no comments to include, please type 'Nil'.	[Text entry]: ________________________________

Table S2. Participant characteristics (vaccinated status is defined as two doses).

	All participants<br>n = 842<br>n, (%)	Vaccinated<br>n = 509 (60.5%)<br>n, (%)	Not vaccinated<br>n = 333 (39.5%)<br>n, (%)
Male	457 (54.3)	279 (61.1)	178 (38.9)
Female*	378 (44.9)	225 (59.5)	153 (40.5)
**Age: median**	58 (IQR 19)	59 (IQR 18)	56 (IQR 22.5)
**Age (years)**			
18 – 49	251 (29.8)	134 (53.4)	117 (46.6)
50 – 69	455 (54.0)	284 (62.4)	171 (37.6)
≥70	136 (16.2)	91 (66.9)	45 (33.1)
**Highest level of education†**			
No formal education/primary school	37 (4.4)	16 (43.2)	21 (56.8)
Secondary school	319 (37.9)	191 (59.9)	128 (40.1)
Vocational/Trade	231 (27.4)	132 (57.1)	99 (42.9)
University	253 (30.0)	170 (67.2)	83 (32.8)
**Annual household income (AUD)**			
<50K	323 (38.4)	203 (62.8)	120 (37.2)
50-100K	217 (25.8)	126 (58.1)	91 (41.9)
100K-150K	92 (10.9)	56 (60.9)	36 (39.1)
>150K	52 (6.2)	34 (65.4)	18 (34.6)
Prefer not to say	158 (18.8)	90 (57.0)	68 (43.0)
**Aboriginal / Torres Strait Islander‡**			
Yes	25 (3.0)	18 (72.0)	7 (28.0)
**English as first language**			
Yes	677 (80.4)	418 (61.7)	259 (38.3)
No	165 (19.6)	91 (55.2)	74 (44.8)
**Location**			
Metropolitan	605 (71.9)	355 (58.7)	250 (41.3)
Regional	237 (28.1)	154 (65.0)	83 (35.0)
**Diabetes Type**			
Type 1	252 (29.9)	132 (52.4)	120 (47.6)
Type 2	557 (66.2)	360 (64.6)	197 (35.4)
Other/Don't know	33 (3.9)	17 (51.5)	16 (48.5)

**Time since diagnosis**			
<1 year	37 (4.4)	24 (64.9)	13 (35.1)
1 – 5 years	106 (12.6)	51 (48.1)	55 (51.9)
5.1 – 10 years	174 (20.7)	91 (52.3)	83 (47.7)
>10 years	525 (62.4)	343 (65.3)	182 (34.7)
**Most recent HbA1c within the past year**			
<7%	139 (16.6)	82 (59.0)	57 (41.0)
7% – 8.5%	334 (39.9)	213 (63.8)	121 (36.2)
8.6 – 10%	148 (17.7)	89 (60.1)	59 (39.9)
>10%	67 (8.0)	43 (64.2)	24 (35.8)
Don't know	150 (17.9)	80 (53.3)	70 (46.7)
**Current diabetes treatment**			
Insulin	279 (33.1)	150 (53.8)	129 (46.2)
Tablets	173 (20.6)	104 (60.1)	69 (39.9)
Injectables (not insulin)	11 (1.3)	5 (45.5)	6 (54.5)
Diet only	15 (1.8)	11 (73.3)	4 (26.7)
Combination of treatments/Other	364 (43.2)	239 (65.7)	125 (34.3)
**Management of diabetes in the past month:**			
Poor	46 (5.5)	26 (56.5)	20 (43.5)
Fair	169 (20.1)	102 (60.4)	67 (39.6)
Good	302 (35.9)	185 (61.3)	117 (38.7)
Very good	237 (28.2)	139 (58.6)	98 (41.4)
Excellent	87 (10.3)	56 (64.4)	31 (35.6)
**Diabetes affect daily activities in last 4 weeks**			
All the time	52 (6.2)	30 (57.7)	22 (42.3)
Most of the time	92 (10.9)	49 (53.3)	43 (46.7)
Some of the time	222 (26.4)	125 (56.3)	97 (43.7)
Not very often	229 (27.2)	146 (63.8)	83 (36.2)
Not at all	246 (29.3)	158 (64.2)	88 (35.8)

Notes: *There was also "Non-binary/other" (n = 7, 0.8%); †There was also "Other" (n = 2, 0.2%); ‡There was also "Prefer not to say" (n = 12, 1.4%). Abbreviations: IQR, interquartile range; AUD, Australian Dollars; K, 1000; HbA1c, glycated haemoglobin.

Table S3. Hierarchical multivariable logistic regression analysis predicting vaccinated status with sociodemographic and clinical characteristics (n = 809).

**Block and variable (reference)**	**B (SE)**	**p-value**	**OR (95% CI)**
Block 1			
**Time since study commencement**	0.01 (0.01)	0.39	1.01 (0.99 – 1.03)
Block 2			
**Age**	0.03 (0.01)	<0.001	1.03 (1.02 – 1.05)
Block 3			
**Diabetes type (Type 1 diabetes)**			
Type 2 diabetes	0.51 (0.23)	0.03	1.66 (1.05 – 2.62)

Notes: Variables entered into the model at each block: Block 1, time since study commencement; Block 2, age; Block 3, diabetes type. Variable categories excluded from analysis: don't know/other (diabetes type). Abbreviations: B (SE), unstandardized coefficient (standard error); OR (95% CI), odds ratio (95% confidence interval).

Table S4. Logistic regression analysis predicting vaccinated status (defined as two doses) with sociodemographic and clinical characteristics.

Category (reference, n)	B (SE)	OR (95% CI)	p-value
**Age (n = 842)**	0.02 (0.005)	1.02 (1.01 – 1.03)	<0.001
**Time since diagnosis (>10 years, n = 824)**			
≤5 years	-0.54 (0.19)	0.58 (0.40 – 0.85)	0.004
5.1 – 10 years	-0.53 (0.18)	0.59 (0.42 – 0.84)	0.003
**Current diabetes treatment (Yes, n = 842)**			
No	0.33 (0.55)	1.40 (0.48 – 4.07)	0.54

Notes: Regression analyses was controlled for time since study commencement. The time since diagnosis variable categories of <1 year and 1 – 5 years were combined for analyses due to low number of responses for the former category. Abbreviations: B (SE), unstandardized coefficient (standard error); OR (95% CI), odds ratio (95% confidence interval).

Table S5. Hierarchical multivariable logistic regression analysis predicting vaccinated status (defined as two doses) with sociodemographic and clinical characteristics (n = 809).

**Block and variable (reference)**	**B (SE)**	**p-value**	**OR (95% CI)**
Block 1			
**Time since study commencement**	0.02 (0.01)	0.052	1.02 (1.00 – 1.04)
Block 2			
**Age**	0.02 (0.01)	<0.001	1.02 (1.01 – 1.03)
Block 3			
**Time since diagnosis (>10 years)**			
≤5 years	-0.33 (0.21)	0.10	0.72 (0.48 – 1.07)
5.1 – 10 years	-0.55 (0.18)	0.002	0.58 (0.40 – 0.82)

Notes: Variables entered into the model at each block: Block 1, time since study commencement; Block 2, age; Block 3, time since diagnosis. Abbreviations: B (SE), unstandardized coefficient (standard error); OR (95% CI), odds ratio (95% confidence interval).

Table S6. Linear regression analysis predicting the Oxford COVID-19 Vaccine Confidence and Complacency Scale –Summary scale score and the Collective Importance and Beliefs about COVID-19 Vaccine subscale' scores with sociodemographic and clinical characteristics.

	Step 1	Step 2			
**Variable (reference, n)**	**Adj. R^2**	**Adj. R^2**	**Δ Adj. R^2**	**B (SE)**	**p-value**
Summary scale					
**Gender (Male, n = 469)**	0.001	0.013	0.012		
Female				1.82 (0.69)	0.01
**Age (n = 473)**	0.00	0.031	0.031	-0.09 (0.02)	<0.001
**Most recent HbA1c (<7%, n = 471)**	0.00	0.009	0.009		
7% – 8.5%				-1.69 (0.96)	0.08
8.6% – 10%				-0.51 (1.11)	0.64
>10%				-1.82 (1.41)	0.20
Don't know				-2.95 (1.17)	0.01
**Diabetes impact on daily activities in the past four weeks (All of the time/Most of the time, n = 473)**	0.00	0.014	0.014		
Some of the time				-2.81 (1.06)	0.01
Not at all/Not very often				-2.53 (0.95)	0.01
Collective Importance subscale					
**English as first language (Yes, n = 703)**	0.004	0.01	0.006		
No				-0.69 (0.29)	0.02
**Diabetes Type (Type 1, n = 679)**	0.004	0.013	0.009		
Type 2				-0.69 (0.26)	0.01
**Diabetes management over the past month (Excellent, n = 703)**	0.004	0.009	0.005		
Poor				0.86 (0.64)	0.18
Fair				1.14 (0.42)	0.01
Good				0.94 (0.39)	0.02
Very good				0.91 (0.40)	0.02

**Diabetes impact on daily activities in the past four weeks (All of the time/Most of the time, n = 703)**	0.004	0.009	0.005		
Not at all/Not very often				-0.71 (0.32)	0.03
Some of the time				-0.40 (0.35)	0.26
Beliefs about COVID-19 Vaccine subscale					
**Gender (Male, n = 625)**	0.003	0.02	0.017		
Female				0.54 (0.16)	0.001

Notes: Regression analyses was controlled for time since study commencement. Diabetes type (don't know/other) were excluded for comparisons between Type 1 and Type 2 diabetes. Abbreviations: Adj. R^2, Adjusted R^2; B(SE), unstandardized coefficient (standard error).

Table S7. Hierarchical multivariable linear regression analysis of the Oxford COVID-19 Vaccine Confidence and Complacency Scale score (n = 467).

Step and variable (reference)	Adj. R^2	Δ Adj. R^2	B (SE)	p-value	sr
Step 1	0.001	0.001			
**Time since study commencement**			0.05 (0.04)	0.26	0.05
Step 2	0.036	0.035			
**Gender (Male)**					
Female			1.37 (0.69)	0.05	0.09
**Age**			-0.08 (0.02)	<0.001	-0.16
Step 3	0.054	0.018			
**Most recent HbA1c (<7%)**					
7% – 8.5%			-1.28 (0.95)	0.18	-0.06
8.6% – 10%			0.21 (1.10)	0.85	0.01
>10%			-1.60 (1.39)	0.25	-0.05
Don't know			-2.26 (1.16)	0.05	-0.09
**Diabetes impact on daily activities in past four weeks (All of the time/Most of the time)**					
Some of the time			-2.93 (1.06)	0.01	-0.13
Not at all/Not very often			-2.14 (0.95)	0.03	-0.10

Notes: Variables entered into the model at each step: Step 1, time since study commencement; Step 2, gender, age; Step 3, most recent HbA1c, diabetes impact on daily activities in past four weeks. Variable categories excluded from analysis: Non-binary/prefer not to say (gender). Abbreviations: Adj. R^2, Adjusted R^2; B(SE), unstandardized coefficient (standard error); sr, semipartial correlation coefficient.

Table S8. Hierarchical multivariable linear regression analysis of the Oxford COVID-19 Vaccine Confidence and Complacency – Collective Importance score (n = 679).

**Step and variable (reference)**	**Adj. R^2**	**ΔAdj. R^2**	**B (SE)**	**p-value**	**sr**
Step 1	0.004	0.004			
**Time since study commencement**			0.03 (0.01)	0.06	0.07
Step 2	0.009	0.005			
**English as first language (Yes)**					
No			-0.66 (0.30)	0.03	-0.08
Step 3	0.019	0.010			
**Diabetes Type (Type 1)**					
Type 2			-0.56 (0.26)	0.04	-0.08
**Diabetes management in past month (Excellent)**					
Poor			0.54 (0.66)	0.41	0.03
Fair			0.87 (0.45)	0.05	0.07
Good			0.78 (0.41)	0.06	0.07
Very good			0.73 (0.42)	0.08	0.07
**Diabetes impact on daily activities in past four weeks (All of the time/Most of the time)**					
Some of the time			-0.35 (0.36)	0.34	-0.04
Not at all/Not very often			-0.51 (0.34)	0.13	-0.06

Notes: Variables entered into the model at each step: Step 1, time since study commencement; Step 2, English as first language; Step 3, diabetes type, diabetes management in past month, diabetes impact on daily activities in past four weeks. Variable categories excluded from analysis: don't know/other (diabetes type). Abbreviations: Adj. R^2, Adjusted R^2; B(SE), unstandardized coefficient (standard error); sr, semipartial correlation coefficient.

Table S9. Linear regression analysis predicting the Oxford COVID-19 Vaccine Confidence and Complacency Scale – Speed of Vaccine Development subscale score with sociodemographic and clinical characteristics.

	Step 1	Step 2			
**Variable (reference, n)**	**Adj. R^2**	**Adj. R^2**	**Δ Adj. R^2**	**B (SE)**	**p-value**
**Gender (Male, n = 673)**	0.004	0.016	0.012		
Female				0.59 (0.20)	0.003
**Age (n = 679)**	0.003	0.038	0.035	-0.03 (0.01)	<0.001
**Annual household income (AUD<50K, n = 679)**	0.003	0.022	0.019		
50K–100K				0.67 (0.25)	0.01
100K–150K				1.17 (0.34)	0.001
>150K				0.88 (0.43)	0.04
Prefer not to say				0.59 (0.28)	0.03
**English as first language (Yes, n = 679)**	0.003	0.036	0.033		
No				-1.24 (0.25)	<0.001
**Location (Metropolitan, n = 679)**	0.003	0.007	0.004		
Regional/rural				0.89 (0.49)	0.07
**Diabetes Type (Type 1, n = 659)**	0.003	0.016	0.013		
Type 2				-0.73 (0.23)	0.001
**Most recent HbA1c (<7%, n = 676)**	0.003	0.009	0.006		
7% – 8.5%				-0.35 (0.29)	0.23
8.6% – 10%				-0.38 (0.34)	0.26
>10%				-0.76 (0.43)	0.08
Don’t know				-0.09 (0.34)	0.01

Notes: Regression analyses was controlled for time since study commencement at step 1. These variables were excluded due to <30 responses; Aboriginal and/or Torres Strait Islander status: non-binary/other gender. Diabetes type (don’t know/other) were excluded for comparisons between Type 1 and Type 2 diabetes. Abbreviations: Adj. R^2, Adjusted R^2; B(SE), unstandardized coefficient (standard error); AUD, Australian Dollars; K, 1000; HbA1c, glycated haemoglobin.

Table S10. Hierarchical multivariable linear regression analysis of the Oxford COVID-19 Vaccine Confidence and Complacency Scale – Speed of Vaccine Development (n = 651).

Step and variable (reference)	Adj. R^2	Δ Adj. R^2	B (SE)	p-value	sr
Step 1	0.03	0.03			
**Time since study commencement**			0.02 (0.01)	0.08	0.07
Step 2	0.078	0.075			
**Gender (Male)**					
Female			0.34 (0.20)	0.09	0.06
**Age**			-0.03 (0.01)	<0.001	-0.15
**Annual household income (AUD<50K)**					
50K–100K			0.45 (0.25)	0.08	0.07
100K–150K			0.75 (0.35)	0.03	0.08
>150K			0.53 (0.44)	0.23	0.05
Prefer not to say			0.34 (0.28)	0.23	0.05
**English as first language (Yes)**					
No			-1.16 (0.26)	<0.001	-0.17
Step 3	0.078	0.00			
**Diabetes Type (Type 1)**					
Type 2			-0.13 (0.25)	0.61	-0.02
**Most recent HbA1c (<7%)**					
7% – 8.5%			-0.26 (0.29)	0.34	-0.04
8.6% – 10%			-0.37 (0.33)	0.26	-0.04
>10%			-0.71 (0.42)	0.09	-0.06
Don't know			-0.62 (0.34)	0.07	-0.07

Notes: Variables entered into the model at each step: Step 1, time since study commencement; Step 2, age, gender, annual household income, English as a first language; Step 3, diabetes type, most recent HbA1c. Variable categories excluded from analysis: Non-binary/prefer not to say (gender); don't know/other (diabetes type). Abbreviations: Adj. R^2, Adjusted $R^{2;}$ B(SE), unstandardized coefficient (standard error); sr, semipartial correlation coefficient; AUD, Australian Dollars; K, 1000; HbA1c, glycated haemaglobin.

Table S11. Linear regression analysis predicting the Oxford COVID-19 Vaccine Confidence and Complacency Scale – Side-Effects subscale score with sociodemographic and clinical characteristics.

	**Step 1**	**Step 2**			
**Category (reference, n)**	**Adj. R^2**	**Adj. R^2**	**Δ Adj. R^2**	**B (SE)**	**p-value**
**Gender (Male, n = 694)**	-0.001	0.004	0.005		
Female				0.36 (0.17)	0.03
**Age (n = 700)**	-0.001	0.049	0.05	-0.04 (0.01)	<0.001
**Current diabetes treatment (Yes, n = 700)**	-0.001	-0.003	-0.002		
No				0.13 (0.59)	0.83
**Diabetes management over the past month (Excellent, n = 700)**	-0.001	0.013	0.014		
Poor				1.10 (0.46)	0.02
Fair				1.16 (0.31)	<0.001
Good				0.78 (0.29)	0.01
Very good				0.81 (0.30)	0.01
**Diabetes impact on daily activities in the past four weeks (All of the time/Most of the time, n = 700)**	-0.001	0.014	0.015		
Some of the time				-0.41 (0.26)	0.12
Not at all/Not very often				-0.80 (0.23)	0.001

Notes: Regression analyses was controlled for time since study commencement. These variables were excluded due to <30 responses: non-binary/other gender. Abbreviations: Adj. R^2, Adjusted R^2; B(SE), unstandardized coefficient (standard error).

Table S12. Hierarchical multivariable linear regression analysis of the Oxford COVID-19 Vaccine Confidence and Complacency Scale – Side-Effects score (n = 694).

	Adj. R^2	Δ Adj. R^2	B(SE)	p-value	sr
Step 1	-0.001	-0.001			
**Time since study commencement**			0.003 (0.01)	0.75	0.01
Step 2	0.047	0.048			
**Gender (Male)**					
Female			0.15 (0.17)	0.36	0.03
**Age**			-0.03 (0.01)	<0.001	-0.21
Step 3	0.064	0.017			
**Diabetes management in past month (Excellent)**					
Poor			0.65 (0.45)	0.15	0.05
Fair			1.04 (0.31)	0.001	0.12
Good			0.68 (0.29)	0.02	0.09
Very good			0.74 (0.29)	0.01	0.09
**Diabetes impact on daily activities in past four weeks (All of the time/Most of the time)**					
Some of the time			-0.44 (0.26)	0.08	-0.06
Not at all/Not very often			-0.58 (0.23)	0.01	-0.09

Notes: Variables entered into the model at each step: Step 1, time since study commencement; Step 2, gender, age; Step 3, diabetes management in past month, diabetes impact on daily activities in past four weeks. Variable categories excluded from analysis: Non-binary/prefer not to say (gender). Abbreviations: Adj. R^2, Adjusted R^2; B(SE), unstandardized coefficient(standard error); sr, semipartial correlation coefficient.

Table S13. Linear regression analysis predicting the Disease Influenced Vaccine Acceptance Scale-Six – Disease Complacency and Vaccine Vulnerability subscale' scores with sociodemographic and clinical characteristics.

	**Step 1**	**Step 2**			
**Category (reference, n)**	**Adj. R^2**	**Adj. R^2**	**Δ Adj. R^2**	**B (SE)**	**p-value**
Disease Complacency subscale					
**Highest level of education (No formal /primary school/secondary school, n =770)**	0.002	0.007	0.005		
Vocational/Trade				-0.51 (0.28)	0.07
University				-0.61 (0.27)	0.03
**Diabetes management over the past month (Excellent, n = 771)**	0.001	0.022	0.021		
Poor				-1.15 (0.61)	0.06
Fair				-1.85 (0.44)	<0.001
Good				-1.29 (0.41)	0.002
Very good				-0.92 (0.42)	0.03
Vaccine Vulnerability subscale					
**Diabetes management over the past month (Excellent, n = 724)**	-0.001	0.017	0.018		
Poor				2.19 (0.73)	0.003
Fair				1.58 (0.51)	0.002
Good				0.57 (0.47)	0.23
Very good				0.69 (0.49)	0.16
**Diabetes impact on daily activities in the past four weeks (All of the time/Most of the time, n = 724)**	-0.001	0.019	0.020		
Some of the time				-0.81 (0.41)	0.05
Not at all/Not very often				-1.47 (0.37)	<0.001

Notes: Regression analyses was controlled for time since study commencement. These variables were excluded due to <30 responses: "other" educational level. Abbreviations: Adj. R^2, Adjusted R^2; B(SE), unstandardized coefficient (standard error).

Table S14. Multivariable linear regression analysis of the Disease Influenced Vaccine Acceptance Scale-Six – Disease Complacency score (n = 770).

	Adj. R^2	ΔAdj. R^2	B (SE)	p-value	sr
Step 1	0.002	0.002			
**Time since study commencement**			-0.02 (0.01)	0.14	-0.05
Step 2	0.007	0.005			
**Highest level of education (No formal /primary school/secondary school)**					
Vocational/Trade			-0.51 (0.28)	0.07	-0.07
University			-0.61 (0.27)	0.03	-0.08
Step 3	0.026	0.019			
**Diabetes management in past month (Excellent)**					
Poor			-1.16 (0.61)	0.06	-0.07
Fair			-1.85 (0.44)	<0.001	-0.15
Good			-1.31 (0.40)	0.001	-0.12
Very good			-0.97 (0.42)	0.02	-0.08

Notes: Variables entered into the model at each step: Step 1, time since study commencement; Step 2, highest level of education; Step 3, diabetes management in past month. Variable categories excluded from analysis: other (highest level of education). Abbreviations: B, unstandardized coefficient; SE, standard error; t, t-statistic; sr, semipartial correlation coefficient; Adj. R^2, Adjusted R^2.

Table S15. Multivariable linear regression analysis predicting the Disease Influenced Vaccine Acceptance Scale-Six – Vaccine Vulnerability. subscale score with sociodemographic and clinical characteristics (n = 724).

	**Adj. R^2**	**ΔAdj. R^2**	**B (SE)**	**p-value**	**sr**
Step 1	-0.001	-0.001			
**Time since study commencement**			-0.01 (0.02)	0.52	-0.02
Step 2	0.032	0.033			
**Diabetes management in past month (Excellent)**					
Poor			1.80 (0.73)	0.01	0.09
Fair			1.35 (0.52)	0.01	0.10
Good			0.38 (0.48)	0.43	0.03
Very good			0.56 (0.49)	0.25	0.04
**Diabetes impact on daily activities in past four weeks (All of the time/Most of the time)**					
Some of the time			-0.80 (0.41)	0.05	-0.07
Not at all/Not very often			-1.31 (0.37)	0.00	-0.13

Notes: Variables entered into the model at each step: Step 1, time since study commencement; Step 2, diabetes management in past month, diabetes impact on daily activities in past four weeks. Abbreviations: Adj. R^2, Adjusted R^2; B(SE), unstandardized coefficient (standard error); sr, semipartial correlation coefficient.
